# Post-operative Hamman’s sign: a case report

**DOI:** 10.1186/s40064-015-1172-7

**Published:** 2015-08-13

**Authors:** John Michael DiBianco, Archana Nair, Richard Williams

**Affiliations:** Department of Internal Medicine, MedStar Harbor Hospital 3001 S. Hanover St., Baltimore, MD 21225 USA; Ross University School of Medicine, Roseau, Commonwealth of Dominica, West Indies

**Keywords:** Hamman’s sign, Pneumothorax, Precordial sounds

## Abstract

**Introduction:**

We provide this brief case report on Hamman’s sign, as a reminder that both pneumomediastinum and pneumothorax can cause sounds that may disclose the abnormal presence of air in these respective locations.

**Background:**

Attributed originally to the observations and report of Louis Hamman in 1937, abnormal sounds may emanate from air in the otherwise quiet mediastinum or pleural space.

**Case presentation:**

Our patient, a 36 year old white male, reported the occurrence of an abnormal “rhythmic clicking” sound emanating from his upper body when lying on his left side, 3 weeks following nasal septal surgery. The patient’s clear report of particularly loud sounds, beginning post-operatively, was corroborated by the patient’s wife. A CT scan of the chest which confirmed the presence of air in the left pleural space.

**Conclusions:**

The presence of a sound, loud enough to be heard at a distance from the patient (corroborated by another individual) is unusual. The value of patient history is underscored by the finding of a pneumothorax, suggesting Hamman’s sign.

## Case description

Our patient, a 36 year-old white man, presented to the emergency department following 12 h of right flank pain radiating to the groin, associated with intractable nausea, that had not responded to hydration and ibuprofen. While generally in good health, except for a history of recurrent nephrolithiasis, he was approximately 3 weeks post-operative from a deviated nasal septum repair. He reported being a current smoker with no personal or family history of pulmonary problems, including: asthma, COPD, cystic fibrosis or alpha-1 anti-trypsinase deficiency. His nasal septal surgery and post-operative course had been uneventful, allowing him to return to his construction job 2 weeks after his surgery. He described no pulmonary complaints, however, on review of systems, he described an audible “rhythmic clicking” sound when lying on his left side. His wife confirmed that the sound first appeared soon after his surgery. Initially, she could hear it up to three feet away, most pronounced when lying in bed. Gradually the sound became quieter and infrequent during the subsequent week.

Physical examination revealed head, neck, chest, cardiac and specifically respiratory exam within normal limits. Attempts to reproduce the sound by palpation or positioning in the left lateral decubitus position were unsuccessful. The abdominal exam identified only right flank and costovertebral tenderness.

**Fig. 1:**
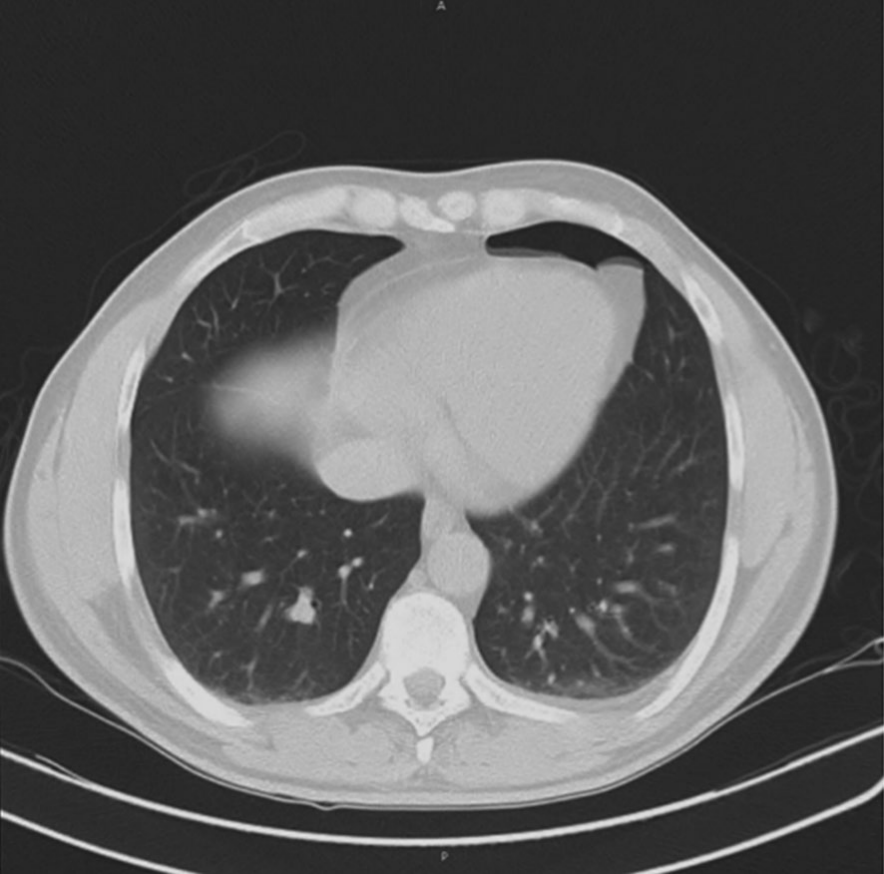
CT chest.

An abdominal and pelvic CT revealed a 5.3 mm left distal ureteral stone with mild hydronephrosis. Proximal abdominal CT scan slices appeared suspicious for a small pneumothorax later confirmed by chest CT which delineated a tiny left anterior basilar pneumothorax, free of mass, nodule, or infiltrate. CT showed no evidence of hilar adenopathy nor bullae (Fig. 1).

Our patient was admitted to the hospital, placed on high flow O_2_ therapy as well as aggressive fluid hydration and tamsulosin 0.4 mg daily. He was discharged on hospital day 2 after a CXR confirmed resolution of the pneumothorax with recommendations to follow up with his primary care physician and urologist.

## Discussion

Laennec and Hamman (1831) and Munden (1918) identified precordial sounds in the setting of mediastinal emphysema. This finding is commonly referred to by the eponym, Hamman’s Sign. An article however, by Roelandt et al. (1969) proposed that many of the sounds attributed to mediastinal emphysema may have actually emanated from left sided cases of pneumothorax. While limited in number, several papers and case reports address this clinical observation and its potential significance (Roelandt et al. 1969; Scadding and Lond 1939; Baumann and Sahn 1992; Wilkinson and Adams 2012; Remmelts 2010. Scadding and Wood appear to be the first authors to describe precordial sounds, specifically, systolic clicks, in the presence of small spontaneous left sided cases of pneumothorax (Scadding and Lond 1939).

Depending on the clinical setting, the presence of precordial sounds, observed or patient reported, should expand the differential diagnosis to include those associated with pneumothoraces in addition to other pulmonary, cardiac and esophageal disorders.

## Evaluation

Our patient likely experienced this left anterior pneumothorax due to the positive pressure ventilation during his nasal septum repair consistent, with the report of the sounds created, being temporally related to the surgery. Approximately 10% of all pneumothoraces are a result of barotrauma (British Thoracic Society Fitness to Dive Group 2003). However, the majority of patients with pneumothorax present with some degree of pulmonary complaints, including: dyspnea and chest pain (British Thoracic Society Fitness to Dive Group 2003). In the absence of large chest wounds with accompanying mediastinal emphysema, it is rare for a pneumothorax to create sound, that is, present with Hamman’s Sign.

Although it cannot be absolutely confirmed that the anterior pneumothorax produced the sounds described by the patient and his wife, its temporal association with surgery, the anterior location of the pneumothorax (adjacent to the pericardium), resolution with disappearance of the pneumothorax and similarity to a rare but previously recognized clinical entity, Hamman’s sign, support that the sound was generated by the pneumothorax.

## Conclusion

The presence of a sound, loud enough to be heard at a distance from the patient (corroborated by another individual) is an unusual and to our knowledge previously unreported finding of pneumothorax. The value of patient history is underscored by the finding of a pneumothorax, suggesting Hamman’s sign.
